# A Comparison of Java, Flutter and Kotlin/Native Technologies for Sensor Data-Driven Applications

**DOI:** 10.3390/s21103324

**Published:** 2021-05-11

**Authors:** Kamil Wasilewski, Wojciech Zabierowski

**Affiliations:** Department of Microelectronic and Computer Science, Lodz University of Technology, ul. Wólczańska 221/223, 90-924 Łódź, Poland; kamil.wasilewskki@gmail.com

**Keywords:** mobile applications, mobile frameworks, performance measures, programming language selection

## Abstract

As a result of the continuous progress and fast-growing popularity of mobile technologies in recent years, the demand for mobile applications has increased rapidly. One of the most important decisions that its developers have to make is the choice of technology on which their application will be based. This article is devoted to the comparison of Java, Flutter, and Kotlin/Native technologies for applications based on processing and analyzing data from sensors. The main elements of the comparison are the efficiency and resource utilization of mobile applications for Android OS implemented in each of the aforementioned technologies.

## 1. Introduction

The choice of method of creating sensor data-driven applications for mobile devices depends on many factors. One of these factors is the workload and thus the time spent on development. Optimization of the execution time is crucial due to the costs of creating and maintaining the implemented application [1]. Certainly, it is necessary to refer to the standards that should be met by such applications, e.g., ISO/IEC 25,010 and ISO/IEC 9126 [2].

In addition, when optimizing the application development time, the device’s energy consumption must be considered alongside its resource utilization [3]. This is important because the target devices are powered by batteries with limited capacity. Numerous studies have been conducted on this issue, including both the impact of execution time [4] and the use of Bluetooth LE for communication with beacons [5] on energy consumption.

In our department, we research systems based on various sensor networks. The results of this research are then used, for example, in medical projects supported by the Polish National Centre for Research and Development (StrategMed program) that are or will be implemented in the industry (e.g., a device that monitors and prevents falls of patients, or an intelligent suite for paramedics). Many of these projects involve the design of custom sensors, such as appropriately integrated accelerometers and gyroscopes, which also have to have much better response times and accuracy than those commercially available. Although mobile solutions may have almost unlimited resources currently, standard smartphones could not be used for our solutions due to the obligatory miniaturization. Therefore, we had to find a solution that would both process the data collected from the sensor network quickly, and yet use so few resources that the target device could be compact. The implemented solution also had to be reliable: delayed or incorrect processing of sensor data may have led to the malfunctioning of the whole system. For example, in the aforementioned application for fall prevention, an excessive delay in data processing can lead to delayed impulse transmission and, consequently, to a patient’s fall.

Corral, Sillitti, and Succi [6] conducted a comparative analysis of the performance of native and hybrid applications using the PhoneGap front-end for one version of the Android OS. In turn, Delía et al. [7] presented a comparative analysis of the performance achieved by mobile applications developed natively and with the use of multi-platform solutions. The authors focused on the implementation time of native applications and the different methods of creating mobile applications for many platforms at once. However, this is not the approach presented in our article, especially regarding Kotlin/Native and Flutter.

In other publications, comparisons of performance, such as Android Native Development Kit (NDK) vs. Dalvik [8,9] were made. The authors conducted various tests, such as numeric calculations (including the Ackerman recursive function), sorting (Hash, Hash2), nested loops, random number generations, and operations on strings. As a rule, it makes sense to take such a set of tests, but in our opinion, they do not fully reflect the most common and relevant operations used when working with mobile applications.

The tests in this publication generally refer to the qualities of the language itself and are not particularly characterized by communication with the outside world. The tests in our article, due to their specificity, refer more to the most common operations (such as object serialization and deserialization, REST requests, and operations on collections, files, or databases) that are performed during the use of mobile applications. However, it should be taken into consideration that other tests can certainly be invented (e.g., a performance test of matrix operations), and do not affect the reliability and correct view of the results obtained by us.

There are many performance-related comparisons in the literature, but these are comparisons of specific application solutions [10] rather than, as in this article, of the basics affecting the final result of the application. This makes existing papers less versatile, and thus their results could be used only in applications that meet very specific conditions. For this reason, the authors of this publication have decided to answer the question of the impact on performance as a result of the use of different programming languages, depending on the operations performed by the target application. In order to conduct the tests, Java was chosen as the native language for creating native applications for Android devices, while Flutter [11,12] and Kotlin/Native [13] frameworks were used for the other solutions.

According to our experience from previous projects and discussions with other teams, the choice of programming technology was usually based on subjective feelings and habits, or even came down to implementing a language because someone knew it beforehand. Our research, which arose during the academic research of the previously mentioned medical projects, gives the opportunity to make an appropriate choice of mobile application technology based on the very specific parameters described in this article, according to requirements.

## 2. Background and Technologies Description

### 2.1. Mobile Application

A mobile application (mobile app) is a software application designed to run on a mobile device such as a smartphone, tablet, or watch [14]. There are countless purposes of using them: communication; social media; news; sending emails; shopping; making payments; watching videos and streaming; playing games.

The number of mobile application downloads has been steadily increasing each year [15]. Increased incomes and the number of active users have resulted in an increasing number of businesses making their services accessible via mobile devices. Applications such as medical ones for patients [16], those supporting farmers [17], and those making life easier for people with disabilities [18] have been developed.

Along with the popularity of these solutions, questions about the performance of developed applications have emerged. Does the application run fast enough and does it not freeze the device during start-up? Are operations performed fast enough? Is the development and testing process short enough to make creating subsequent builds not too burdensome for the programmers? It is important that the creation process is not too long and therefore too expensive.

### 2.2. Mobile Platforms

**Figure 1:**
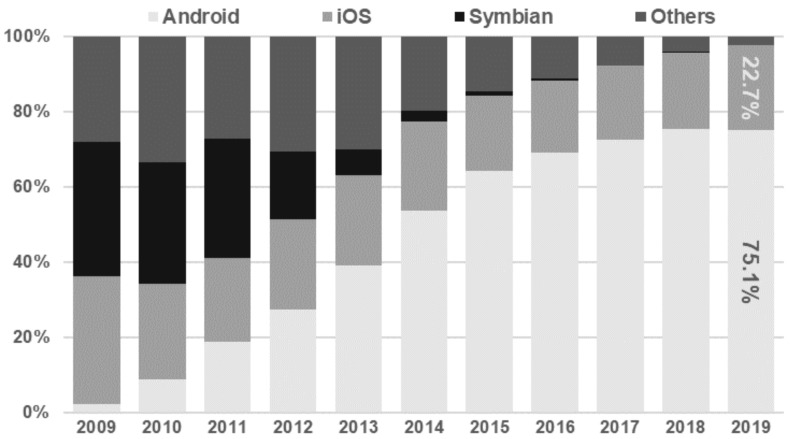
Worldwide market share of mobile operating systems.

Currently, Android (Google) and iOS (Apple) have almost 98% of the worldwide market share of mobile operating systems, which, in practice, limits the choice of the target platform for the new applications to them. The importance of other formerly common operating systems such as BlackBerry or Symbian is marginal (Figure 1).

Due to the existence of two significant mobile platforms, developers are required to decide at an early stage of development whether the final solution will be native, web-based, or hybrid [19,20].

### 2.3. Application Types

#### 2.3.1. Native

Native applications are those that have been developed and designed to run on a specific platform. The source code is compiled to obtain executable code. This is a process similar to the one used in traditional applications.

When an application is ready to be distributed, it is uploaded to platform-specific application stores, where it is then audited to ensure that the application meets the requirements of the target platform.

An important characteristic of native applications is that, due to their very high compatibility with a particular platform or even a specific device, they can use all peripherals provided by a given system, including sensors [21,22].

#### 2.3.2. Web

Mobile web applications are designed to run in the device’s browser. They are developed using standard web technologies, so they are nothing more than web pages made with HTML, CSS, and JavaScript [23]. The advantage of this solution is undoubtedly their extreme portability. Such applications will work correctly on all devices with implemented web browsers that are compatible with W3C standards. However, there are some limitations that derive from the principles of the operating system architecture. Access to the functionality of the device and system is limited, so this mobile application type will not be suitable for all solutions [24].

#### 2.3.3. Hybrid

Hybrid applications are a certain combination of the solutions described above. They are a mixture of web technologies on the client’s side that also run in a platform-specific container. This gives an advantage: the code can be easily transferred between platforms. At the same time, thanks to using API dedicated to a specific platform, hybrid applications also have access to many functionalities of both the device and the given operating system [25].

### 2.4. Technologies

#### 2.4.1. Android OS

Android was designed by Android Inc. (Palo Alto, CA, USA), whose initial concept was to develop a complex operating system for digital cameras [26]. The idea has evolved due to the small market for these devices. Thus, the new goal was to produce a solution dedicated to mobile devices, competing with Windows Mobile and Symbian.

In June 2005, Android Inc. was purchased by Google for more than USD 50 million. The first commercial version of the Android was released 3 years later, in September 2008.

In May 2019, Google announced at its annual conference for developers, “Google I/O”, that there were more than 2.5 billion active Android devices globally [27] and that the number of programs available for this platform was greater than 3.6 million.

#### 2.4.2. Java

Java is a class-based, object-oriented, general-purpose programming language. Its origins date back to 1991, when James Gosling of Sun Microsystems decided to implement a simple, small, and platform-independent language. The first public implementation was released as Java 1.0 in 1996. In 2010, Sun Microsystems was purchased by Oracle, which has been responsible for Java development since then [28].

Java is currently one of the most popular languages in the world and thus has one of the largest global communities. This community provides a great number of free libraries and ready-to-use solutions.

#### 2.4.3. Flutter

Flutter is an open-source mobile application development framework created by Google in 2017. It is used to develop applications for Android and iOS, and is also the primary method of creating applications for a successor of Android, Google Fuchsia.

Mobile applications based on the Flutter framework are written in the Dart language [12]. The biggest advantage of this solution is that it allows the use of the same code base for both iOS and Android applications. Another important feature is the simplicity of building a user interface that is created from the code level.

#### 2.4.4. Kotlin/Native

Kotlin/Native is a Kotlin module developed by JetBrains that is capable of compiling a single code base for iOS and Android applications.

The first early version of the framework was released at the beginning of 2017 and the stable version was released in September 2018. A large community also takes part in the development of the technology, preparing libraries that expand the possibilities of Kotlin/Native.

Applications developed with the use of this framework are written in the Kotlin language. One codebase allows for the implementation of versions for all popular platforms, rather than only mobile ones [13].

## 3. Materials and Methods

In order to conduct the tests, three mobile applications were developed, one for each of the technologies: Java (1.8), Flutter (1.5.4-hotfix.2), and Kotlin/Native (1.3.40). Both the performance and the resource utilization of the device were measured using the Android Profiler tool built into the Android Studio (3.4.1). Before each test, an Android full garbage collection was manually forced (in the case of Flutter, Dart’s garbage collection was forced, too).

### 3.1. Test Rig

All applications and the REST server have been developed, built, and deployed on a PC with the following specification:

- Motherboard: Asus ROG Maximus XI Hero;
- CPU: Intel Core i9-9900k (5.0 GHZ);
- RAM: 2 × 8 GB (dual-channel, 4000 MHZ);
- GPU: Asus ROG Strix GeForce RTX 2080 Ti OC;
- SSD: Samsung 970 EVO 1TB;
- OS: Windows 10 Pro (October 2018 Update).

In addition, the above-mentioned computer has been running an Android Virtual Machine with the following specification:

- Emulated device: Google Pixel 3;
- OS: Android 9.0 Pie (API 28);
- CPU cores: 4;
- RAM: 2 GB.

### 3.2. Test Assumptions: Build Time and Application File Size

In order to reduce the size of the applications, they have been built to a minimized (shrunk and obfuscated) version. Gradle (5.5) was responsible for the building process.

### 3.3. Test Assumptions: Start-Up of the Application and Idle Usage of RAM

The effect of both tests is the average result of 10 attempts. RAM usage was recorded 30 s after the application was launched.

### 3.4. Test Assumptions: Collection Operations

In order to test collection operations, the simplest implementations of the list were used: ArrayList for Java and Kotlin/Native, and List for Flutter. The single item in each test was an object containing one double and one string variable; both variables were assigned a schematically generated value.

The sequential adding test consisted of adding 10,000 items one by one to the end of an initially empty list. The sequential reading test consisted of obtaining 10,000 items one by one, starting from the beginning of the list filled with 10,000 items. The result of both tests is the average time taken to perform a single operation (writing/reading an item).

The random reading test consisted of obtaining 10,000 items, one by one, from random places on the list of 100,000 items. The result of the test is the average time taken to obtain a single item based on a random index.

The random removal test consisted of removing 10,000 items, one by one, from random places on the list of 100,000 items. The result of the test is the average removal time of a single item based on a random index.

The result of the filtering test is the average duration of the filtering of the list containing 10,000 items, based on 10,000 attempts.

The result of the sorting test is the average duration of the sorting list containing 10,000 shuffled items, based on 10,000 attempts.

### 3.5. Test Assumptions: REST—GET and POST

The REST server was developed using the Spring framework. In order to guarantee the shortest response to the request, the server was deployed locally. 

Tests of REST requests consisted of 10,000 GET and POST requests. The GET response body and the POST request body included a JSON object containing one long, one double, and three string variables; all variables were assigned a schematically generated value. The result of the tests is the average response time for a single request.

### 3.6. Test Assumptions: Database Operations

All technologies allow the use of the SQLite database, natively supported by Android. In the case of the Kotlin/Native application, it was required to use the SQLDelight library.

The database used in the tests consists of one table containing 2 integer-type columns and 3 text-type columns.

The result of the INSERT test is the average time needed to add a single record to the initially empty “USERS” table, using the INSERT statement, based on 1000 attempts.

The result of the SELECT ALL test is the average time needed to obtain all the records from the “USERS” table that was initially filled with 10,000 records (using the SELECT statement without WHERE clause), based on 1000 attempts.

The result of the SELECT ONE, UPDATE ONE, and DELETE ONE test is the average time needed to perform a single operation (obtaining one item using the SELECT statement with the WHERE clause, updating a single item using the UPDATE statement, or deleting one item using the DELETE statement), based on 1000 attempts. In each case, the table was initially filled with 10,000 records.

### 3.7. Test Assumptions: Serialization and Deserialization

In order to test serialization and deserialization, an object with the same structure was implemented in each application. The object contained one long, one double, and three string variables; all variables were assigned a schematically generated value.

The result of each test is the average time needed to perform a single operation (serializing an object to JSON, or deserializing an object from JSON) based on 10,000 attempts.

### 3.8. Test Assumptions: File Operations

In all tests, 100 MB files were used. The device’s internal memory was used as storage.

The save and remove test results are the average time needed to perform a single operation (saving a file and removing a file, respectively) based on 100 attempts.

The file read test result is the average time needed to read a file based on 100 attempts.

## 4. Results

### 4.1. Build Time and Application File Size

**Figure 2:**
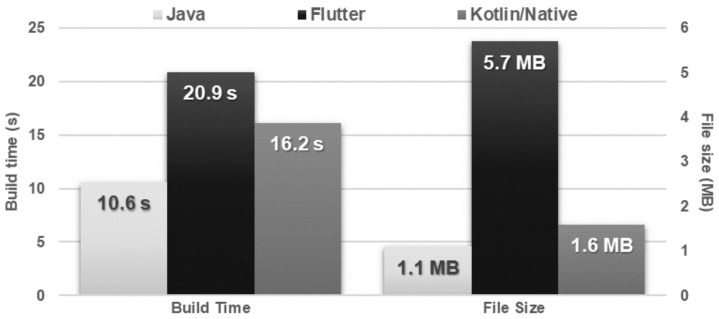
Average application build time and size of the installation file.

Java required nearly half as much time to build the application as Flutter and approximately 35% less time than Kotlin/Native. In addition, the size of the Java application was more than five times smaller than Flutter and more than 30% smaller than Kotlin/Native (Figure 2).

### 4.2. Start-Up of the Application and Idle Usage of RAM

**Figure 3:**
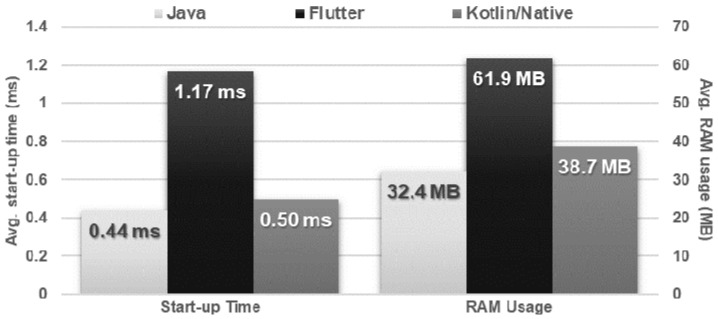
Average start-up time and average idle usage of RAM.

The results of the Flutter application differed negatively from the results of the other applications. It took more than double the time to start up and was the only application that was not smooth and immediate. In addition, the Flutter application required almost twice as much memory as Java when idle, and 60% more than Kotlin/Native (Figure 3).

### 4.3. Collection Operations

**Figure 4:**
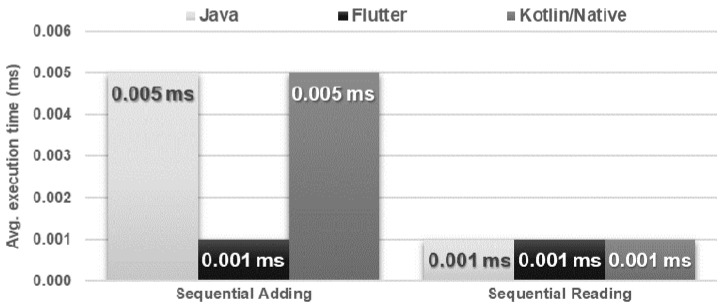
Average adding and reading times of a single item.

**Figure 5:**
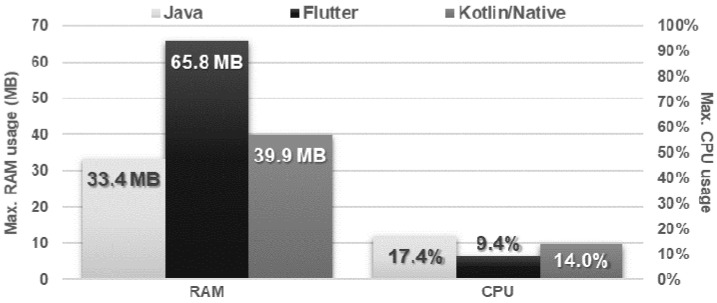
Maximum usage of RAM and CPU during sequential adding and sequential reading.

**Figure 6:**
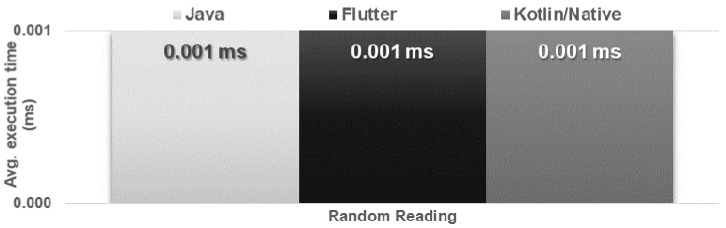
Average time of reading an item at a random point in the list.

**Figure 7:**
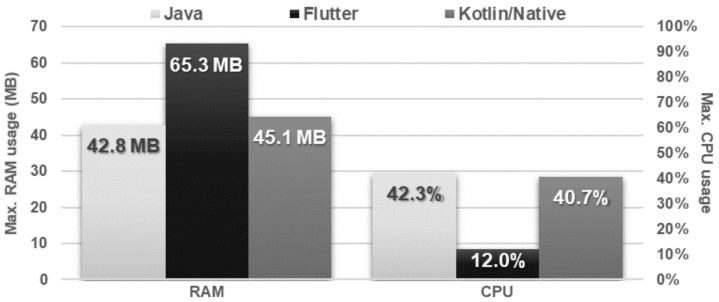
Maximum usage of RAM and CPU during random item reading.

**Figure 8:**
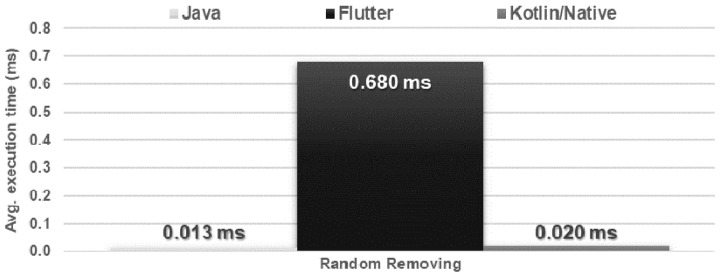
Average removal time of a single item from a random place in the list.

**Figure 9:**
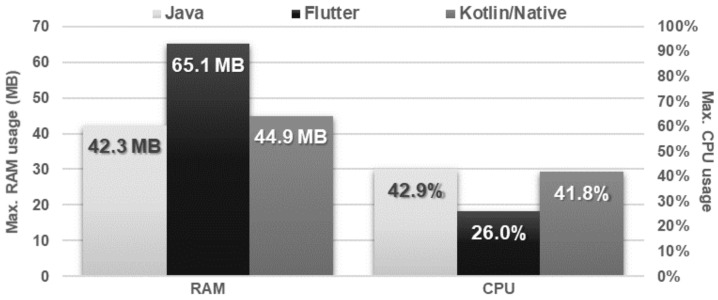
Maximum usage of RAM and CPU during random item removal.

**Figure 10:**
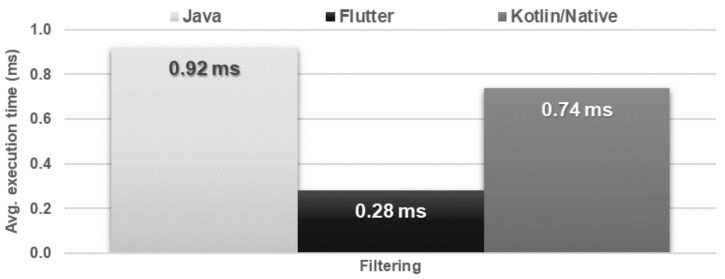
Average time of list filtering.

**Figure 11:**
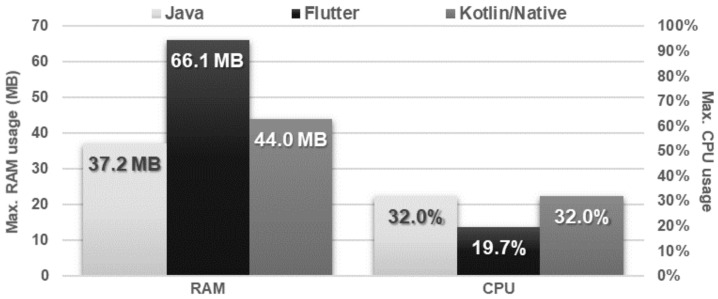
Maximum usage of RAM and CPU during list filtering.

**Figure 12:**
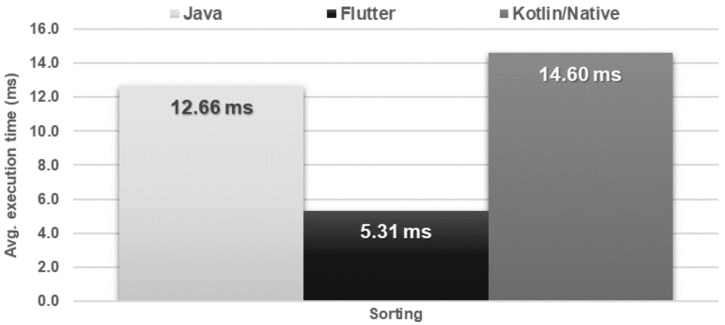
Average time of list sorting.

**Figure 13:**
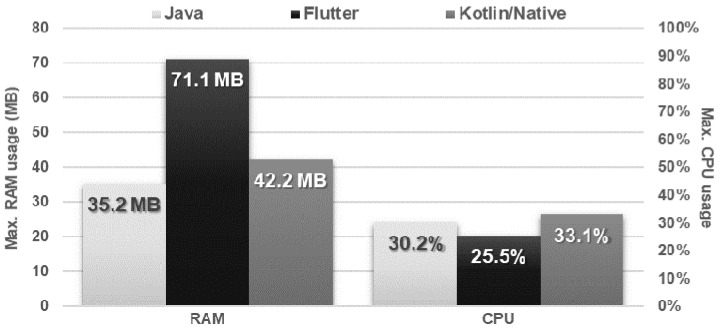
Maximum usage of RAM and CPU during list sorting.

The obtained results do not allow explicit selection of the most effective technology for collection operations (Figure 4, Figure 5, Figure 6, Figure 7, Figure 8, Figure 9, Figure 10, Figure 11, Figure 12, and Figure 13). In three out of six tests, the shortest execution time was obtained by the Flutter application (Figure 4, Figure 10, and Figure 12). However, in the random removal test, Java was over 52 times faster, and Kotlin/Native was 34 times faster (Figure 8). In the remaining two tests, the execution time was the same for each of the technologies (Figure 4 and Figure 6). The Kotlin/Native and Java applications were exceptionally similar both in terms of execution times and resource utilization. Flutter had the lowest CPU usage in each test but also used the most RAM (Figure 5, Figure 7, Figure 9, Figure 11, and Figure 13).

### 4.4. REST—GET and POST

**Figure 14:**
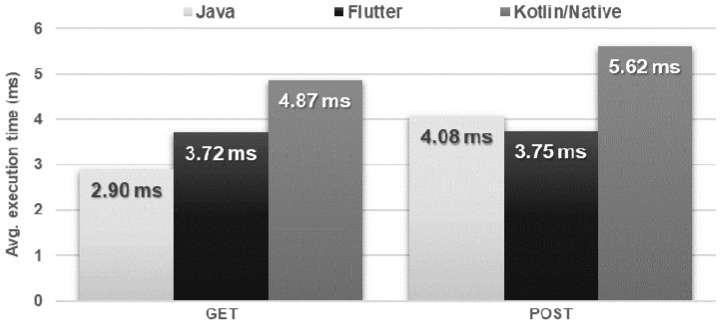
Average response time for a single REST request.

**Figure 15:**
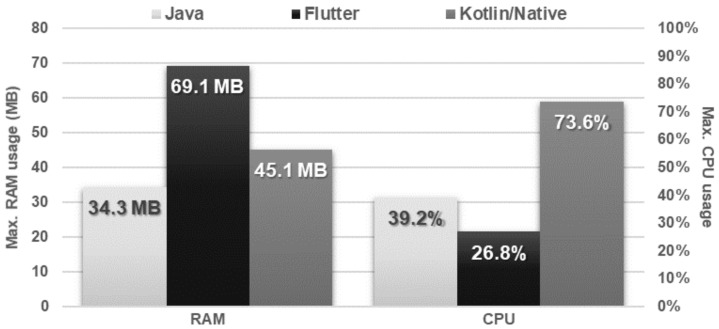
Maximum usage of RAM and CPU during request execution.

The Kotlin/Native application negatively differed in terms of the time of execution of both tested types of REST requests. In addition, it used more CPU power than the other applications. The results of the Flutter and Java applications were similar, except for the use of RAM, where Flutter required almost twice as much at the peak (Figure 14 and Figure 15).

### 4.5. Database Operations

**Figure 16:**
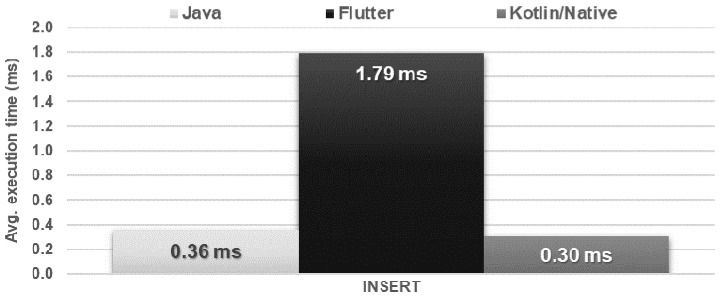
Average time to insert a single record.

**Figure 17:**
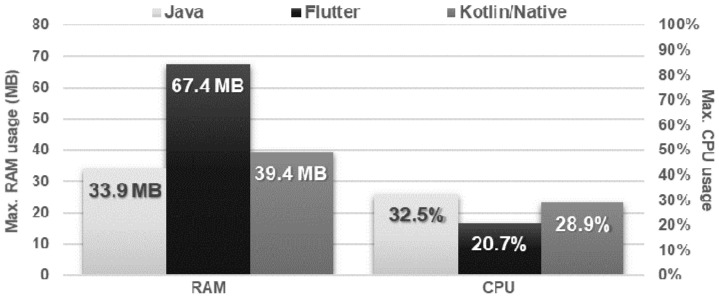
Maximum RAM and CPU usage when inserting the records.

**Figure 18:**
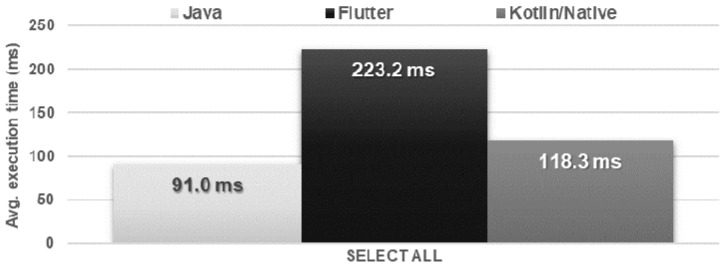
Average time to obtain all the records.

**Figure 19:**
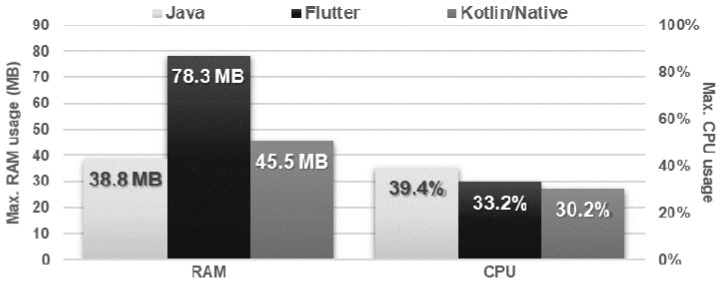
Maximum RAM and CPU usage during the obtaining of all the records.

**Figure 20:**
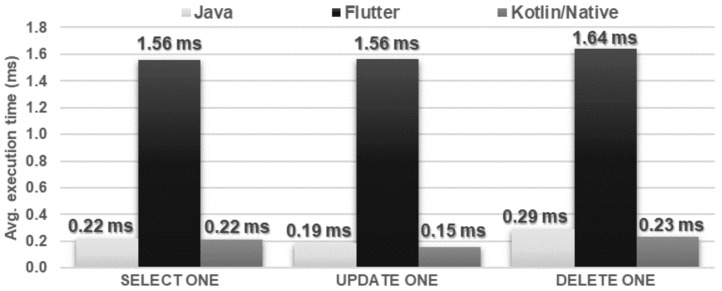
Average time of execution of the SELECT, UPDATE, DELETE operations.

**Figure 21:**
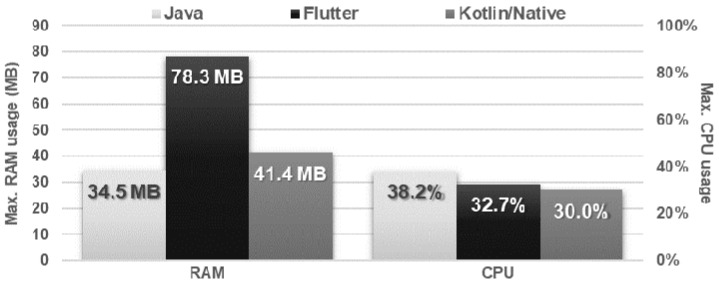
Maximum usage of RAM and CPU during the SELECT ONE, UPDATE ONE, DELETE ONE tests.

The Flutter application showed the worst performance in database operations. It needed several times more time to perform each of the operations and used approximately twice as much memory as the other applications. Kotlin/Native and Java applications achieved very similar, good results. The use of the processor was almost the same for each technology (Figure 16, Figure 17, Figure 18, Figure 19, Figure 20, and Figure 21).

### 4.6. Serialization and Deserialization

**Figure 22:**
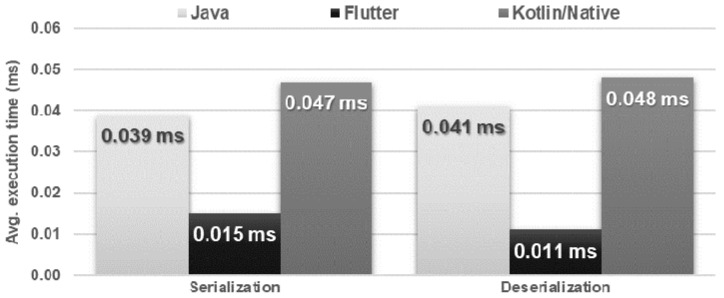
Average times for serializing and deserializing an object.

**Figure 23:**
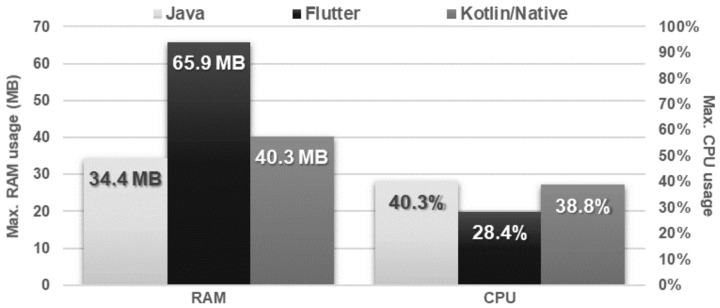
Maximum usage of RAM and CPU during the test.

The Flutter application in both tests was approximately four times faster in terms of execution time, but, at the same time, used most of the RAM. Other applications not only needed more time to perform both operations but also used more CPU power (Figure 22 and Figure 23).

### 4.7. File Operations

**Figure 24:**
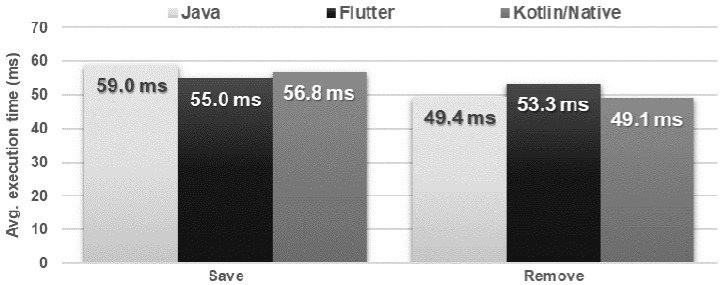
Average times for saving and removing a file.

**Figure 25:**
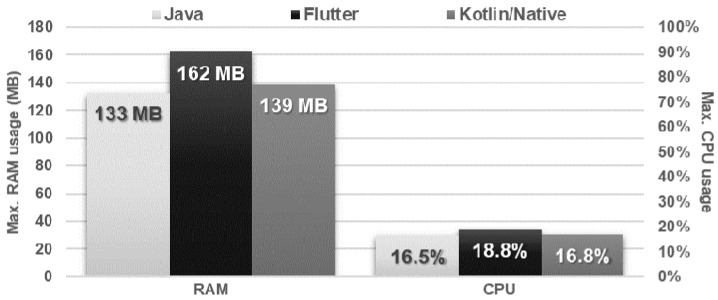
Maximum usage of RAM and CPU during file saving and deleting.

**Figure 26:**
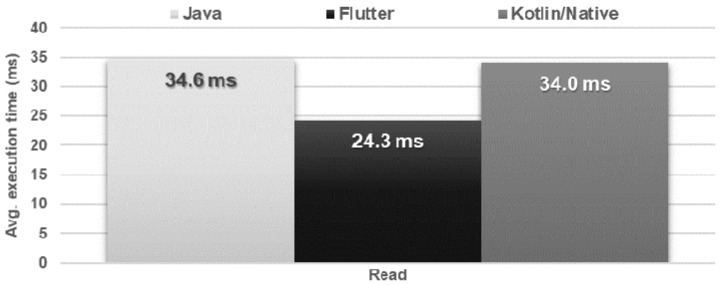
Average times for file reading.

**Figure 27:**
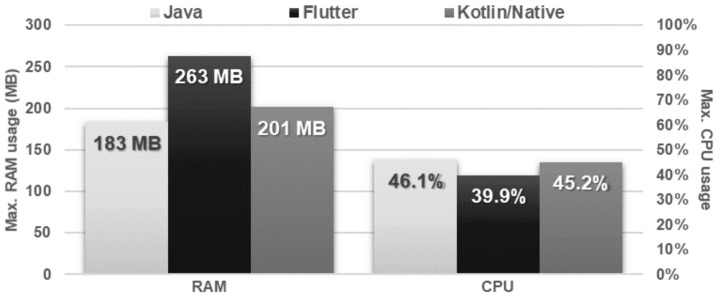
Maximum usage of RAM and CPU during file reading.

The time and amount of resources needed to save and delete the file were very similar for each application (Figure 24 and Figure 25). In the case of reading the file, the Flutter application turned out to be the fastest, but used much more memory in relation to the other applications (Figure 26 and Figure 27).

## 5. Discussion

Considering the time of execution for each operation, the most noteworthy is the Flutter application, which, despite obtaining the best time results in the largest number of tests, was outclassed in the database operations tests where the competitive applications were as much as 10 times faster.

The resource utilization for each application is also interesting. In the case of RAM usage, the Flutter application was the worst and the Java application was the best. The opposite was the case for CPU usage, where the best results were achieved by the Flutter application, and the Java and Kotlin/Native applications had similar, worse results.

The results of the Java applications in terms of the time needed to perform each of the operations were the most stable, and in none of the tests were they significantly different from those achieved by other applications. At the same time, it had the smallest RAM usage.

It should always be remembered that the real workload of the executed application must be taken into consideration when planning. The presented results must be considered differently for an application that saves individual pieces of data to a database, and for when huge amounts of data are generated from sensors for further processing, as is the case with some mobile application solutions [16]. It is different when operations on the files are sporadic, and it is different when it is a process of creating and saving large amounts of even small files.

Finally, it should also be pointed out that both Flutter and Kotlin/Native are still in the early stages of development and, therefore, it is to be expected that the next versions should solve some problems. However, this does not mean that developers should be afraid of creating applications using these technologies. Their extensive documentation makes them easy to learn, and the growing community around them will help with any problems that may arise.

## 6. Conclusions

The following conclusions emerge from the conducted tests. Wherever there is not a concern to save resources and where the execution time is of primary importance, Flutter seems to be a natural choice, particularly, for certain queuing operations, sorting, filtering, serialization, or operations on the files. In some of these tests, Flutter has also proven to be better in terms of CPU usage. The tests also indicate that Flutter should not be used in applications that heavily depend on database operations. The same caution should be taken when using both Java and Kotlin/Native, especially because of some anomalies in the language which can and do affect its creation process [29].

After a period of deliberation, it can be concluded that such results could be expected. In principle, this is true. However, to put forward a thesis and prove it is different, as the authors of this article have indicated.

To conclude, it is important to be aware of both the limitations and advantages of each solution, in order to be able to choose the best solution after a good analysis of the problem. However, experience is always needed to achieve this effectively.

The results of our research are universal, and so will be helpful for anyone who wants to develop an application that collects and processes data from any sensor network. In order to make a final decision, apart from the obtained results, only the information about the most frequently performed operations by the designed mobile application is needed.

This article provides answers to the questions not yet supported by the literature and makes a significant contribution to the development of mobile applications.

## Data Availability

Not applicable.
